# From traditional to innovative: implications of cranial non-metric traits in personal identification

**DOI:** 10.1007/s00414-025-03462-w

**Published:** 2025-03-03

**Authors:** Andrea Palamenghi, Antonio Aragon-Molina, Giulia Caccia, Debora Mazzarelli, Sofia Alemanno, Ruggero Donida Labati, Fabio Scotti, Vincenzo Piuri, Carlo Pietro Campobasso, Cristina Cattaneo, Danilo De Angelis, Daniele Gibelli

**Affiliations:** 1 Laboratorio Di Antropologia e Odontologia Forense, Sezione Di Medicina Legale, Dipartimento Di Scienze Biomediche Per La Salute, LABANOF, Università Degli Studi Di Milano, Via L. Mangiagalli 37, 20133 Milan, Italy; 2 Laboratorio Di Anatomia Funzionale Dell’Apparato Stomatognatico, Dipartimento Di Scienze Biomediche Per La Salute, LAFAS, Università Degli Studi Di Milano, Via L. Mangiagalli 31, 20133 Milan, Italy; 3 Department of Computer Science, Università Degli Studi di Milano, Milano, Italy; 4 Department of Experimental Medicine, University of Campania “Luigi Vanvitelli”, Naples, Italy

**Keywords:** Compound frequencies, Likelihood ratio, Probabilities, Cranium, Unidentified remains, Forensic anthropology

## Abstract

In forensic anthropology, personal identification is mainly performed through a qualitative assessment and comparison of morphological bone and dental features between antemortem and postmortem data. Although non-metric traits have been traditionally considered as individualizing features, their potential has been limitedly investigated. Indeed, frequencies of variants can represent an additional tool to provide probabilities and likelihood ratios that an individual presents a combination of traits, hence quantifying a possible identification. This study investigates the potential of cranial non-metric traits as individualizing features in personal identification, and it describes the application of this probabilistic approach to a sample from a mass fatality which occurred in 2015. 119 crania of males were assessed for scoring 35 non-metric traits by presence and absence. For each cranium, the compound frequencies of independent traits, probabilities and likelihood ratios that a cranium presents a specific blend of traits were calculated. Over 70% of the likelihood ratios exceeded 1,000,000, providing extremely strong evidence that a specific set of traits corresponds to a cranium. Probabilities to find an individual with the set of traits within a group of 528 people (corresponding to the recovered bodies for this case) were extremely low (e.g., 0.006 people out of 528). The considerably high likelihood ratios and low probabilities suggest that combinations of cranial non-metric traits are extremely specific to the single individual, hence they represent valuable individualizing features. Despite this approach does not seem immediately applicable for the resolution of this case because of the dearth of appropriate antemortem images, collecting cranial non-metric frequencies may be worth of further investigation as a supplementary tool to screen potential identities and provide quantitative evidence to the investigators and the judge.

## Introduction

Morphological traits of the skeleton represent elements of variability between individuals and play a pivotal role in personal identification of unidentified skeletal remains through the comparison of antemortem (AM) and postmortem (PM) data. To do so, forensic anthropologists look for corresponding markers to prove that the remains under study belong to the missing person of interest.

Traditionally, personal identification relies on qualitative assessments and comparisons to find concordant and discordant traits between antemortem (AM) and postmortem (PM) records [1, 2]. However, forensic anthropology has increasingly focused on enhancing quantitative analyses for various tasks [3–13], including personal identification. In this direction, the interest in applying a quantitative approach to morphological identifiers for identification has recently grown, especially regarding variable features of the skeleton [14]. The first studies on this approach considered frequencies of traumatic and pathological features in contemporary skeletal populations [15, 16], although it has been noted that such features may be too common to confirm an identification when only evaluated as present/absent [16]. Watamaniuk and Rogers [17] suggested that frequencies of morphological variants on the margin of thoracic vertebrae could be used calculate the strength of the identification, hence of the match. Independent frequencies can be multiplied together to calculate the probability that an individual in a group presents a specific combination of traits. Other features that may seem suitable for this approach are therefore non-metric traits, whose frequencies can be calculated in different population groups [14]. Non-metric traits are intrinsic skeletal variants that present as accessory tubercles, foramina, ossicles, often with an unknown function and origin [18, 19]. Unlike metric traits (e.g., length, diameter), which can be quantified and measured, non-metric traits are evaluated on a qualitative basis and are typically classified as either present or absent, or they are assessed based on the degree of their expression. Over time, anthropologists have mainly investigated non-metric traits to infer biological distance between population groups and kinship in archaeological populations [20]. Most often, researchers have focused on presenting the incidence without examining further potential of the traits. In contrast, a sound knowledge of these variants represents a powerful tool that can be applied to several investigations, including clinical medicine and surgery [21–23], trauma analysis [19], and personal identification [24]. The general assumption is that non-metric traits potentially represent individualizing features that differ among individuals, although little research has been carried out in this sense. Following this research avenue, Palamenghi et al. [24] introduced the potential of cranial non-metric traits for personal identification purposes. By multiplying the frequencies of 12 traits in a sample of the CAL collection [25, 26], the study provided compound frequencies between 1 × 10^− 6^ and 1 × 10^− 9^, which supports the individualizing potential of these features when considered in combination. However, independence of the traits was not tested.

The abovementioned studies about frequencies of morphological bone features and variants specifically aim at providing a statistical framework to personal identification based on skeletal evidence. Furthermore, posterior probabilities and likelihood ratios in the analysis of skeletal remains lay the ground for a statistical basis applied to morphological observations, which can support the evidentiary value of the qualitative assessment in court [27–29]. Likelihood ratios (LR) are statistical tools to communicate the evidentiary value of an assessment, which are commonly employed in DNA comparisons [1]. When applied to personal identification in forensic anthropology, the likelihood ratios represent a mean to express the probability that an individual present specific features if the identity corresponds to that individual, divided by the probability that an individual present such features if the identity does not correspond [1]. This statistical mean thus allows to express the strength of an identification, similarly to DNA comparisons. Known frequencies of skeletal features in a population can thus be used to provide a statistical basis to morphological evaluations. Following previous studies [13, 30], Cappella et al. [31] recorded the frequencies of facial nevi and generated likelihood ratios with values that could provide from moderate to strong and extremely strong support to the hypothesis of an identification, according to the guidelines of the Association of Forensic Science Providers (AFSP) [32].

In forensic anthropology, personal identification is the final task of the analysis of unidentified remains, which can be straightforward when proper material is available. However, forensic anthropologists have been recently facing the challenges posed by specific conditions, where identification may require additional time or can hardly be achieved. This is the case of the victims of migration, a plight that has been concerning forensic scientists for over 20 years [33, 34]. Southern European countries specifically are familiar with this phenomenon, where countless of shipwrecks occur daily in the Mediterranean Sea, with over 10,000 dead and missing migrants in 10 years and incredibly low identification rates [35–37]. It is estimated that over 60% of the victims remain unidentified [36], which perpetrates the suffering of the families looking for them and exacerbates the consequences of the ambiguous loss [38–41]. In the attempt to identify bodies and human remains from this context, forensic pathologists and anthropologists struggle with several hindrances, including the possible limited use of primary identifiers, such as DNA, fingerprints and odontology [34, 42–46]. As a consequence, forensic scientists need to find novel strategies to expand the set of identifiers, which may be eventually considered as reliable as the primary ones [34, 47]. An additional concern is the possible requests by judges to quantify the assessments [14], in an attempt to overcome the current limitations of morphological identification. In addition to the evidentiary value that can be presented in court, assigning a quantitative value to an identification could significantly strengthen the evidence, helping anthropologists achieve identifications “beyond reasonable doubt.” This would not only advance the discipline but also protect the rights of the victims and of their families to identity and to know the fate of their loved ones, respectively [38–40, 48, 49].

This study expands the existing research line on the role of cranial non-metric traits in personal identification by including a sample of crania from an ongoing identification project carried out by the University of Milan [34, 41, 50]. The goal is to investigate frequencies and individualizing potential of an expanded set of traits in this sample, by analyzing compound frequencies, likelihood ratios, and probabilities of a cranium displaying a specific pattern within a group of individuals. This research is part of the FRANTIC project (Frequencies of Anatomical Traits for Identification), funded by the European Union’s Next Generation EU initiative, which seeks to establish cranial non-metric traits as reliable identifiers. Integrating this method with traditional techniques may enhance identification efforts with a statistical support and foster the development of new tools in forensic anthropology for personal identification. Even though the project does not aim to develop a new identification method, further investigating cranial non-metric traits could improve our understanding on these variants that have been traditionally reported as individualizing features.

## Materials and methods

The study sample includes 119 disarticulated crania which were recovered in the Mediterranean Sea after a shipwreck, for which the LABANOF (Laboratory of Forensic Anthropology and Odontology) of the University of Milan is carrying out a project to achieve positive identification of the victims [41, 50–53]. The data included in this study were drawn as part of the anthropological data collection for the examination of the crania, the elaboration of the biological profile and future identification. All applications on these remains are part of the attempt at identifying the victims; all data included therefore were collected with the unique scope of reaching identification.

Skeletal sex estimation [54] revealed 83% of the crania are males and 17% are females. However, current morphometric methods for sex estimation exhibit low accuracy in this population [50], which differs significantly from standard reference groups [55]. DNA analysis was also performed, confirming that all the crania belong to males.

All crania presented visible cranial sutures were visible, allowing for the assessment of Wormian bones. Age-at-death estimation was based on dental development including the maxillary third molars [56], assessed through intraoral radiographs carried out with a portable dental X-ray unit (intraoral sensor (EZ Sensor 1.5, Vatech, Hwaseong, Republic of Korea) and X-ray tube (model Rextar, Poskom Co. Ltd., Goyang, Republic of Korea). When third molars were missing, age-at-death was estimated according to spheno-occipital synchondrosis closure [57]. According to circumstantial evidence and the antemortem information collected so far, the victims originally came from Sub-Saharan African countries [41]. This consideration was taken into account when selecting the age range based on third molar development [56].Given the lack of definitive provenance, of suitable reference populations and of reliable methods to estimate age-at-death of an adult cranium, these age estimations should be regarded as approximate (Table 1).

**Table 1:** Age-at-death distribution according to third molar development and spheno-occipital closure

Third molar and spheno-occipital stage	Age (years)	*N*° of crania
D	14–18	1
E	14–19	2
F	15–20	6
G	16–23	16
H	> 17	80
Fusing spheno-occipital (missing molars)	16–23	2
Fused spheno-occipital (missing molars)	> 19	12

A set of 35 cranial non-metric traits (25 bilateral and 10 unpaired) was assessed by side and scored as present or absent for each cranium (Table 2). The traits, selected from a longer list of variants that are usually assessed as part of the skeletal analysis at LABANOF, were chosen by two authors with an extensive experience in this kind of assessment, focusing on the features that presented the most clear and unequivocable definition based on the available literature on the topic [19, 58, 59]. When absent, the feature was scored as 0, whereas 1 indicated the presence, except for the palatine bridge which was scored according to degree of closure and the hypoglossal canal which followed the number of canals observed. Therefore, a binary code was generated for each cranium, in which a value of 0/1 was given to each trait- except for the palatine bridge and hypoglossal canal that were scored as 0/1/2 and 1/2/3/4. Data were collected on an Excel spreadsheet to facilitate the following calculations of compound frequencies, probabilities and likelihood ratio of a cranium to present a specific pattern of traits.

**Table 2:** Description of the cranial non-metric traits included in the study

Category	Trait	Description	Bilateral	Median	Scoring
Accessory ossicles and bipartite bones	1. Wormian bone on coronal suture	Accessory bone along the coronal suture	x		0/1
	2. Wormian bone on sagittal suture	Accessory bone along the sagittal suture		x	0/1
	3. Wormian bone on lambdoid suture	Accessory bone along the lambdoid suture	x		0/1
	4. Bregmatic bone	Accessory bone at bregma		x	0/1
	5. Lambdoid bone	Accessory bone at lambda		x	0/1
	6. Inca bone	Accessory bone at the squamous part of the occipital bone		x	0/1
	7. Ossicle at asterion	Accessory bone at asterion	x		0/1
	8. Bipartite parietal bone	Partial or complete division of the parietal squama	x		0/1
	9. Squamo-parietal bone	Accessory bone at the squamous suture	x		0/1
	10. Epipteric bone	Accessory bone at pterion	x		0/1
	11. Os japonicum	Division of the zygomatic bone with one or more accessory sutures	x		0/1
	12. Bipartite temporal squama	Partial or complete division of the temporal squama	x		0/1
Sutures	13. Metopism	Persistent suture along the midline of the frontal bone		x	0/1
	14. Metopic fissure	V- or W-shaped remnant of the metopic suture		x	0/1
	15. Supranasal suture	Suture located in the glabellar region, generated by the ossification processes that occur with the closure of the nasal part of the frontal suture		x	0/1
	16. Mendosa suture	Suture that divides the occipital squama into an upper and lower part	x		0/1
	17. Infraorbital suture	Suture between the maxilla and the greater wing of the sphenoid bone, in the lower part of the orbit	x		0/1
Foramina and notches	18. Parietal foramen	Foramina on one or both sides of the sagittal suture (in the region of the obelion)	x		0/1
	19. Occipital foramen	Foramen occipital squama at the level of the inion or slightly above it		x	0/1
	20. Nasal foramen	One or more foramina perforating the nasal bones	x		0/1
	21. Supraorbital foramen and accessory foramina	Foramen and accessory foramine (reduced in size with respect of the main one) on the superior margin of the orbit	x		0/1
	22. Supraorbital notch	Notch on the superior margin of the orbit	x		0/1
	23. Accessory infraorbital foramen	Accessory foramen located immediately adjacent to the main foramen	x		0/1
	24. Mastoid foramen	Foramen on the posterior surface of the mastoid	x		0/1
	25. Accessory zygomatic foramen	Accessory foramen on the zygomatic bone	x		0/1
	26. Foramen of Vesalius	Foramen anterior to the oval foramen in the medial portion of the greater sphenoid wing	x		0/1
Tori and tubercles	27. Palatine torus	Bony protuberance along the median palatine suture		x	0/1
	28. Maxillary torus	Bony protuberance on the palatal surface of the molar alveoli	x		0/1
	29. Precondylar tubercles	Thickenings on the anterior margin of the occipital foramen	x		0/1
	30. Pharyngeal tubercle	Tubercle located anterior to the foramen magnum		x	0/1
	31. Zygomatic tubercle	Bony tubercle on the lower margin of the zygomatic process or the zygomaticomaxillary suture	x		0/1
Others	32. Posterior condylar canal	One or several foramina posterior to the greater palatine foramen	x		0/1
	33. Frontal grooves	Single or multiple grooves of varying length and depth, located obliquely and laterally on the external surface of the frontal bone	x		0/1
	34. Palatine bridge	Bony spicules that partly or completely connect portions of the palatine bones	x		0 (absent); 1(incomplete); 2 (complete)
	35. Hypoglossal canal	There can be a single, or multiple canals divided by bony laminae	x		1 (single canal); 2 (double canal); 3 (triple canal); 4 (quadruple canal)

The frequencies of the present and absent traits were then calculated, both for the right and the left sides for bilateral traits. A preliminary test was conducted to assess the independence of the features. First, the Pearson correlation coefficient was calculated to determine if there was a significant relationship between the variables. The results showed correlation values of|r| < 0.4, indicating a weak correlation between the features. Second, a chi-squared test of independence was performed to assess pairwise feature relationships. This test allowed us to identify which features exhibit significant dependence, with a threshold set for determining independence. As expected, most of the bilateral features showed a high chi-squared value, indicating a strong dependency between them. The following features were removed from the estimation of the compound frequency to ensure the independence of the remaining features:

- F.1 Wormian bone on coronal suture R.
- F.3 Wormian bone on lambdoid suture R.
- F.5 Lamboid bone.
- F.6 Inca bone.
- F.7 Ossicle at asterion R.
- F.9 Squamo-Parietal bone.
- F.10 Epipteric bone R.
- F.11 Os Japonicum R.
- F.14 Metopic fissure.
- F.16 Mendosa suture R.
- F.17 Infraorbital suture R.
- F.21 Supraorbital foramen and accessory foramina R.
- F.23 Accesory Infraorbital foramen R.
- F.24 Mastoid foramen L.
- F.25 Accessory zygomatic foramen R.
- F.26 Foramen of Vesalius R.
- F.28 Maxillary torus R.
- F.29 Precondylar tubercles R.
- F.31 Zygomatic tubercle.
- F.32 Posterior condylar canal R.
- F.33 Frontal grooves R.

Following this, for each cranium, the frequencies of the traits were multiplied together to obtain a compound frequency (CF), which was then used to calculate the likelihood ratio (LR) expressing the likelihood that a cranium present a specific pattern of non-metric traits using: LR_pattern_= $\:\frac{1}{CF}.$ The evidentiary support of the likelihood ratios (LR) was categorized based on the AFSP classification [32] (Table 3). Probabilities to present a pattern of traits were calculated as well, by multiplying the CF of each cranium and 528, i.e., the number of bodies recovered for the event [41] from which the sample of crania was drawn.

**Table 3:** Support categories for LR values as in the ASFP classification [32]

LR value	Support category
1	no support
> 1–10	weak support
10–100	moderate support
100-1.000	moderately strong support
1.000–10.000	strong support
10.000–1.000.000	very strong support
> 1.000.000	extremely strong support

## Results

The most present frequent traits in the sample were the mastoid foramina (92%), followed by the single hypoglossal canal (84%), nasal foramina (80%) and the supraorbital notch (75%). The bipartite parietal bone and temporal squama were never observed, with a frequency of 0%. Wormian bones on the lambdoid suture, followed by Wormian bones on the coronal suture and squamoparietal bone, were the most common accessory bones, whereas the rarest were the bregmatic bone, the os japonicum and Wormian bones on the sagittal suture. The supranasal suture was the most common accessory suture (42%), followed by the infraorbital suture (24.5%), whereas metopism and sutura medonsa, and metopic fissure, were expressed by 3% and 1% of the sample, respectively. Apart from the aforementioned foramina and notches, parietal foramina and the foramen of Vesalius were recorded in 60% and 70% of the cases, respectively. Supraorbital and accessory supraorbital foramina, accessory zygomatic foramina were quite common as well, as they were recorded in half of the sample (52–59%). The rarest foramen was the occipital foramen (45%). Tori and tubercles were most represented by maxillary torus (39%) and zygomatic tubercle (63%). Posterior condylar canals were recorded in more than half of the sample (59–63%), whereas the frontal grooves were less extensively represented (38–40%). Palatine bridges were most commonly absent (47–52%). As already mentioned, the single hypoglossal canal was the most represented. Notably, the double canal was recorded more frequently on the left side (21%) than in the right side (7%). Triple and quadruple forms were overall underrepresented (1–2%) (Table 4).

**Table 4:** Frequency of the presence of the 35 variants considered in the study

Trait	Side	
	Right	Left
1. Wormian bone on coronal suture	0.29	0.29
2. Wormian bone on sagittal suture	0.08	
3. Wormian bone on lambdoid suture	0.40	0.50
4. Bregmatic bone	0.01	
5. Lambdoid bone	0.13	
6. Inca bone	0.03	
7. Ossicle at asterion	0.19	0.17
8. Bipartite parietal bone	0.00	0.00
9. Squamo-parietal bone	0.22	0.20
10. Epipteric bone	0.10	0.08
11. Os japonicum	0.04	0.03
12. Bipartite temporal squama	0.00	0.00
13. Metopism	0.03	
14. Metopic fissure	0.01	
15. Supranasal suture	0.42	
16. Mendosa suture	0.03	0.03
17. Infraorbital suture	0.23	0.26
18. Parietal foramen	0.71	0.68
19. Occipital foramen	0.45	
20. Nasal foramen	0.79	0.82
21. Supraorbital foramen and accessory foramina	0.52	0.59
22. Supraorbital notch	0.79	0.72
23. Infraorbital foramen	0.33	0.29
24. Mastoid foramen	0.94	0.91
25. Accessory zygomatic foramen	0.58	0.52
26. Foramen of Vesalius	0.61	0.62
27. Palatine torus	0.30	
28. Maxillary torus	0.39	0.39
29. Precondylar tubercle	0.21	0.18
30. Pharyngeal tubercle	0.71	
31. Zygomatic tubercle	0.64	0.61
32. Posterior condylar canal	0.63	0.59
33. Frontal grooves	0.38	0.40
34. Palatine bridge		
Absent	0.52	0.47
Incomplete	0.26	0.30
Complete	0.22	0.22
35. Hypoglossal canal		
Single	0.92	0.76
Double	0.07	0.21
Triple	0.00	0.01
Quadruple	0.02	0.02

Scoring the traits by presence or absence produced a 60-cypher binary code representing the combination of variants in a single cranium (e.g., 000000000000000000000010001101010011001111000000011100001111). All the binary codes were found to be unique, as no code was repeated with the exact same digits, hence every code exclusively belongs to one individual.

**Fig. 1:**
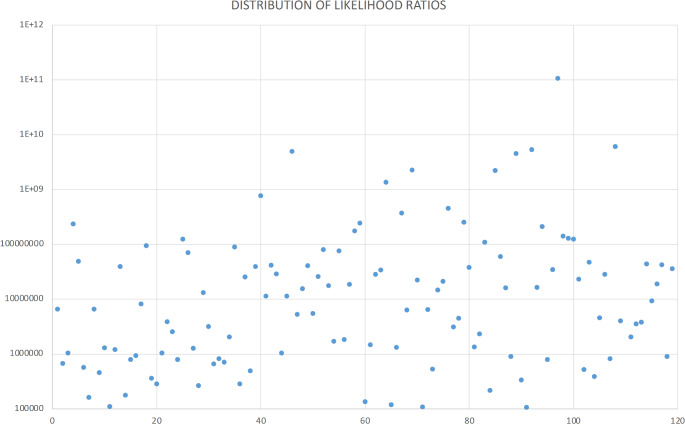
Distribution of likelihood ratios of a cranium to present a specific set of non-metric traits

Based on independent traits, compound frequencies ranged between 1.06963E^− 05^ and 9.36092E^− 12^, with most of the frequencies (81%) between the order of magnitude of 1E^− 06^ and 1E^− 08^. Overall, extremely high values of LR were obtained (Fig. 1). The lowest LR was 93,490, whereas the highest was 1.06827E^+ 11^. Most of the LR values (74%) could be classified within the AFSP support category of extremely strong, as they were all above 1,000,000, while the remaining 26% were classified as very strong support. Even the lowest LR value indicates that it is over 90,000 times more likely that a specific cranium presents the observed pattern of traits within the sample.

**Fig. 2:**
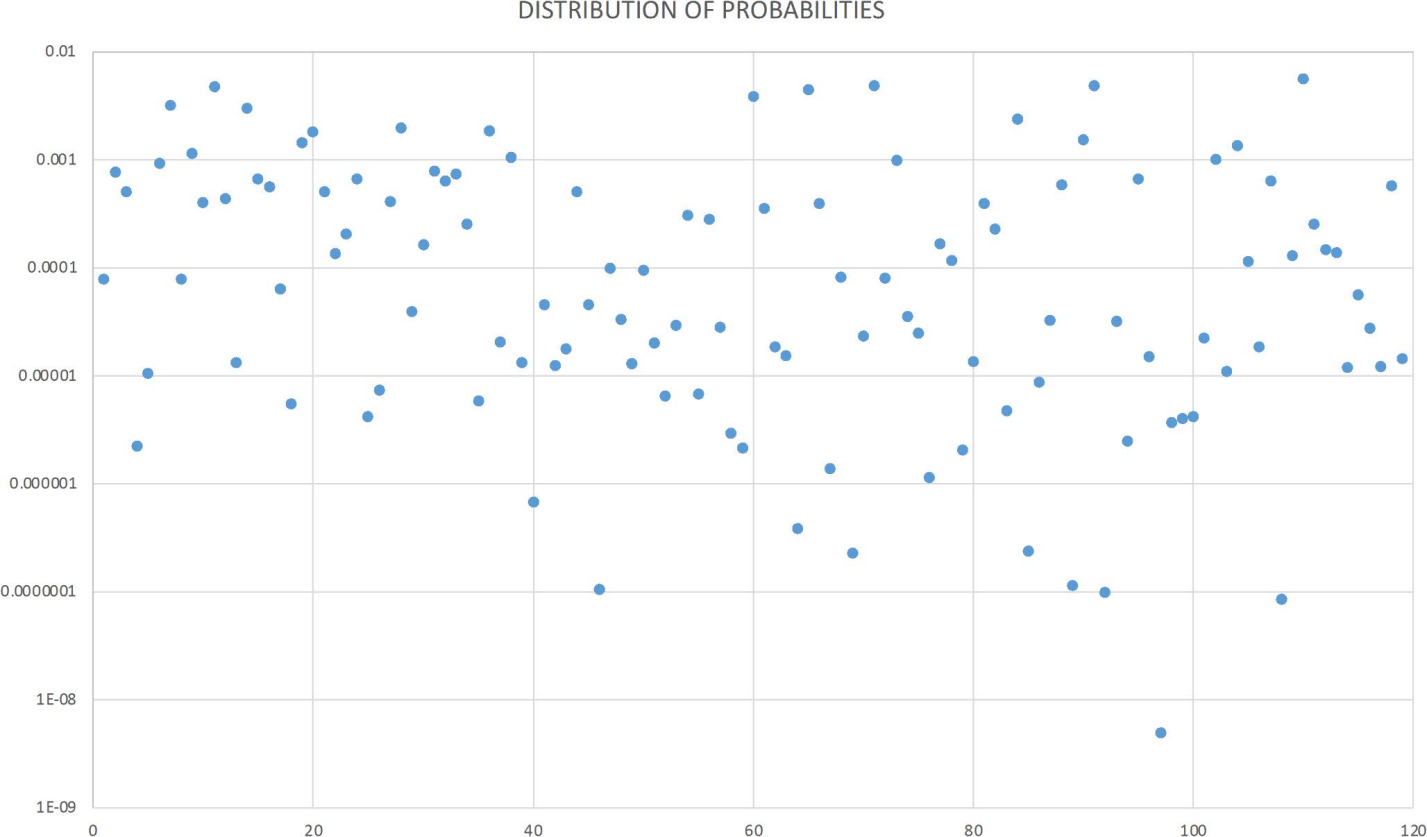
Distribution of probabilities to find a cranium with a specific set of traits in a group of 528 people

Following Watamaniuk and Rogers [17], probabilities of a cranium to present a blend of variants within the group of 528 victims were calculated. The highest and lowest probabilities were 5.65E^− 03^ and 4.94E^− 09^, respectively. The probabilities were almost equally distributed between the order of magnitude of 10^− 4^ and 10^− 5^ (Fig. 2). For example, the compound frequency of 1.07E^− 05^ resulted in probability of the traits occurring together of 0.0056, hence 0.006 people would be expected to present the combination of variants in a group of 528 missing persons.

## Discussion

The uniqueness of so-called secondary identifiers has not been extensively investigated, yet they may prove valuable tools to achieve identification when the application of primary identifiers is hampered. In addition, known frequencies of skeletal traits are suitable tool to build a statistical framework for the identification process, which is needed both to bolster the power of morphological features and to provide the strength of the evidentiary values resulting from qualitative assessments [13, 14, 17, 24, 27, 29, 31]. As a result, non-metric traits can serve this purpose when their frequencies are drawn from a selected population or from a sample that needs to be identified. This study extended this research line providing data on a subset of remains from a forensic humanitarian case, whose identification is still on-going [41, 50, 55]. In a preliminary test on Italian crania [24], only 12 variants were assessed, resulting in higher compound frequencies, the majority of which were between 10^− 6^ and 10^− 7^. Moreover, independence of traits was not tested. Conversely, the present study expanded the set of traits to 35 variants, which led to a considerable improvement in terms of low compound frequencies, hence of low probabilities and high likelihood ratios, even after removing 21 features from the compound frequency. Therefore, the results indicate that the probability to find an individual with the blend of traits is, again, considerably low. Even the highest compound frequency indicates that that there are 0.006 people out of 528 that present a specific combination, which is highly indicative of an identifying potential of the variants. The calculation of the likelihood ratios (LR) further supports the uniqueness of these traits: the large majority of LR obtained from this set of crania were above 1,000,000, which is the highest threshold that indicates an extremely strong support [32] of the hypothesis that a pattern of cranial traits belongs to a specific cranium. The same quantitative approach was recently tested on the pattern of facial nevi [13, 31]. In a test on 1039 individuals, the likelihood ratios based on the compound frequencies were mostly classified as extremely strong support LR values were obtained, whereas the LR based on the individual pattern mainly provided moderately strong support. However, the codes from facial nevi included from 12 to 24 digits, which possibly reduced the identifying potential of the combinations. In contrast, the cranial codes included 60 digits from 35 traits, providing a richer combination, which resulted in exclusive codes for each cranium, extremely low probabilities and extremely strong likelihood ratios.

However, this study has some limitations. A recent reappraisal of secondary identifiers stated that rather than establishing the uniqueness of traits in the general population, one should focus on the context for which identification is needed [60]. Especially in an open disaster, defining the uniqueness of an evidence may be particularly challenging, as several individuals may share specific features. Therefore, to fully understand the consistency and reliability of the observations, and the significance of the individualizing potential presented here, the next analyses will include the whole sample of isolated crania from the identification project. The main limitation to this approach as an additional tool for identification is the current unavailability of AM material suitable for comparison, such as images from CT scan examinations. However, especially in Western countries, the widespread use of CT scan technology for early diagnosis and treatment of trauma or pathology is steadily increasing [61–63], resulting in the availability of these scans as ante-mortem material for personal identification [64–66]. Therefore, this may be of limited use in low resource settings [67], whereas it may be explored with more promising results when appropriate AM is available. However, detecting cranial non-metric traits from 3D models can be challenging [53], highlighting the need for further studies to enhance data collection and interpretation. Observing cross-sectional slices may yield more accurate data, but this approach requires further investigation. Despite this approach may not seem immediately applicable to this disaster, it may be investigated further for displaced and local missing persons that may have been hospitalized or had access to healthcare, and for which AM images could be available. Times are not mature yet to use this approach in casework as libraries of reference data on the distribution of cranial non-metric traits in specific populations should be built to finetune and understand the real individualizing potential of the variants as quantitative tools for identification. An initial effort in this area was made by Christensen and Hatch [1], who developed a repository containing frequencies of anatomical variants from radiological images. Despite some restraints, the recording, analysis and elaboration of frequencies of non-metric traits may represent a valuable additional tool to screen potential candidates within a pool of identities to be conclusively verified with further data [24]. In the future, analyzing hospital CT scans to map and record an individual’s distinct anatomical variations could pave the way for identifying suspects by matching postmortem scans with corresponding anatomical features. Given that automatic analyses of skeletal structures are expanding in clinical and forensic studies [68–71], such advancements may not be completely out of reach and may contribute to significantly expand research and practice of personal identification.

One may never know the origin and type of data that would lead to identification, therefore further research on known populations may benefit the possible development of this novel tool. Regardless of the practical applicability, this study provided for the first time data on frequencies and correlation of non-metric traits in a modern group of individuals from Sub-Saharan countries, for which, to the best of our knowledge, there is a complete dearth. Eventually, the proposed approach does not want to be a standalone identification method or a replacement to well-established identifiers, rather an additional tool to screen potential identities and provide quantitative evidence to the investigators and the judge.

## Conclusions

This study showed that combinations of independent cranial traits occur with substantially low probabilities and high likelihood ratio in an individual, which reinforces the individualizing potential of these traits. Therefore, cranial non-metric traits may provide an additional tool to be used in the screening of possible identities. Further studies will address data collection on traits and frequencies in known populations to fully understand the real potential and possible implementation of this tool in the practice.

## Data Availability

The data presented in this study are available from the corresponding author upon reasonable request.
